# Editorial: Transgenerational Effects of Parental Physical and Mental Illnesses on Their Young Offspring's Adjustment: A Psychosomatic Perspective

**DOI:** 10.3389/fpsyt.2022.901177

**Published:** 2022-04-29

**Authors:** Eliana Tossani, Maria Cristina Verrocchio, Georg Romer, Giulia Landi

**Affiliations:** ^1^Department of Psychology “Renzo Canestrari”, University of Bologna, Bologna, Italy; ^2^Laboratory of Psychosomatics and Clinimetrics, Department of Psychology, University of Bologna, Bologna, Italy; ^3^Department of Psychological, Health and Territorial Sciences, University “Gabriele d'Annunzio” of Chieti-Pescara, Chieti, Italy; ^4^Department of Child and Adolescent Psychiatry, University Hospital Münster, Münster, Germany

**Keywords:** parental illness, intergenerational transmission, young offspring psychosocial adjustment, psychosomatic impacts of parental illness on children and adolescents, parental mental illness, parental physical illness, parental chronic illness, young carers

Children and adolescents of parents with a chronic illness are at increased risk of negative outcomes, including mental and physical health problems, poorer health-related quality of life, educational, and employment difficulties that persist well into adulthood (1–4). Parental chronic illness has wide-ranging impacts on several aspects of young offspring's life such as emotional dysregulation, internalizing and externalizing problems as well as stress-related somatic disorders, and weakened immune responses (3–5). Compared to their peers with “healthy” parents, young offspring of parents with chronic illnesses often experience emotions of shame and guilt, isolation, stigma, and perception of lacking social support (6). Children of parents with a chronic illness also tend to assume responsibilities associated with caring for their parents and are referred to as young carers (7, 8). Not only parental illness itself but also the level of caregiving responsibilities, experiences, and tasks have been associated with poorer outcomes in offspring (9). Hence, research must target the welfare of young offspring of parents with chronic illness.

The papers in this Research Topic examined several of these issues by drawing attention to a lack of research on the transgenerational impact of parental chronic illness on young offspring. Three manuscripts in this collection focused on the impact of parental mental illness on their young offspring (Glaus et al.; Petrovic and Stevovic; Sell et al.). Sell et al. investigated parental illness related-coping as a relevant factor associated with offspring mental health in a German sample of parents with mental illness (*n* = 195) and their offspring aged 4–18 years (*n* = 290). Results of this study indicate that a coping style characterized by religiosity and quest for meaning was associated with fewer internalizing and externalizing problems as well as a lower odds of a mental disorder in the offspring. On the contrary, a coping style characterized by depressive processing was associated with increased internalizing problems in the offspring. In a second manuscript, Petrovic and Stevovic focused on the intergenerational transmission of violence on a sample of adults with schizophrenia or psychotic disorders from Montenegro. Patients in a forensic context having committed violet behaviors (*n* = 20) were compared to patients who did not commit violent behaviors (*n* = 51), and to a control group of healthy controls (*n* = 72). Results revealed that the two groups of patients with a psychiatric diagnosis reported more often to have lived with mentally ill parents and greater exposure to childhood abuse and violence. The presence of parental alcohol abuse, in particular, was significantly higher in the group of patients who committed violent behaviors and revealed to be a plausible mechanism for the intergenerational transmission of violence. Finally, Glaus et al. examined the relationship between maternal trauma history on subsequent offspring somatization and psychopathology. This longitudinal study was conducted on a Swiss sample of mother-toddler dyads (*n* = 64) who were later assessed when offspring had a mean age of 7 years. The presence of interpersonal violence-related post-traumatic stress disorders in mothers was positively correlated with maternal somatization when the offspring were toddlers. Maternal somatization severity at baseline further predicted both maternal reports of child somatization and child thought problems when the offspring were school-aged. This study ultimately underlined the international transmission of somatization in the context of interpersonal violence and related maternal post-traumatic stress disorders.

Two manuscripts in this collection examined the impact of parental cancer on their young offspring (Inhestern, Bultmann et al.; Inhestern, Johannsen et al.). The first is a systematic review of 18 studies conducted by Inhestern, Bultmann et al. which revealed that the prevalence rates of cancer patients having children ranged from 14 to 24.7% and that between 2.5 and 34% of young offspring of parents with cancer report substantial psychosocial burden. The second manuscript (Inhestern, Johannsen et al.) explored patients with cancer (*n* = 78) reporting on their young offspring (*n* = 117) psychosocial adjustment. After the cancer diagnosis, some parents reported that their children were more self-confident, responsible, comfortable in social situations, and improved their school performance. On the other hand, parents also reported increased clinginess and irritability in their offspring as well as higher levels of sadness, fear, withdrawal, difficulties concentrating, and sleeping problems. Ultimately, the quality of life of parents with cancer was positively correlated with that of their offspring.

One manuscript in this collection examined the impact of both parental physical and mental illness on their offspring. Through a longitudinal study conducted on a representative sample of youth (*n* = 1,266) in central Norway, Kaasbøll et al. examined the mediational role of offspring attachment style in the link between parental chronic illness when offspring were adolescents and internalizing symptoms when offspring were young adults. Results revealed that attachment to fathers mediated the relationship between maternal chronic illness in adolescence and internalizing symptoms in young adulthood, while attachment to both mothers and fathers mediated the relationship between paternal chronic illness in adolescence and internalizing symptoms in young adulthood. Separate analyses based on offspring's gender indicated that these results were only significant in female offspring. This study provides an important contribution to potential mediating and moderating mechanisms in the pathways between parental chronic illness and internalizing symptoms in adolescents as they transition into young adulthood.

Finally, in the last manuscript of this collection Lesinskiene discussed current mental health services for offspring adjustment to parental mental illness in Lithuania. The importance of appropriate long term systematic programs and family-focused care in adult psychiatric hospitals was highlighted.

The manuscripts reported in this special issue are a much needed advancement in the exploration of the intergenerational transmission of mental and physical health from parents to young offspring, especially from a psychosomatic perspective. Future studies following a biopsychosocial approach should be conducted including various genetic, individual, family, and environmental risk and protective factors [e.g., (10)] for young offspring living in families with a chronically ill parent. In particular, little is known about the psychosomatic longitudinal mechanisms by which parental chronic illness impacts the next generation. Enhancing our knowledge on this topic might ultimately improve the development and delivery of effective interventions for families experiencing parental chronic illness.

## Author Contributions

ET and GL: conceptualization and writing—original draft preparation. ET, GL, MV, and GR: writing—review and editing and supervision. All authors have read and agreed to the published version of the manuscript.

## Conflict of Interest

The authors declare that the research was conducted in the absence of any commercial or financial relationships that could be construed as a potential conflict of interest.

## Publisher's Note

All claims expressed in this article are solely those of the authors and do not necessarily represent those of their affiliated organizations, or those of the publisher, the editors and the reviewers. Any product that may be evaluated in this article, or claim that may be made by its manufacturer, is not guaranteed or endorsed by the publisher.
